# Computational and Experimental Modeling of Interfaces and Joints in Advanced Materials

**DOI:** 10.3390/ma19081553

**Published:** 2026-04-13

**Authors:** Rafał Grzejda

**Affiliations:** Faculty of Mechanical Engineering and Mechatronics, West Pomeranian University of Technology in Szczecin, 19 Piastow Ave., 70-310 Szczecin, Poland; rafal.grzejda@zut.edu.pl

## 1. Introduction

The importance of properly finished surfaces of machine components and their joints has increased significantly in recent years [1,2,3,4,5]. The aim is to optimize processes in order to guarantee quality and safety standards for components with the least possible impact on the environment [6,7]. One of the strategies being implemented is the introduction of new materials and their combinations. In this context, interfaces and joints between different components are of great importance, as they affect the weight, mechanical properties and production costs of the structures manufactured [8].

In practice, there are two types of joints: non-detachable and detachable. Among non-detachable joints, welded [9,10], riveted [11,12], or press-fit [13,14] joints are most commonly used, while in the case of detachable joints, bolted [15,16], keyway [17,18], or pivoted [19,20] joints are used. They are made not only of steel or metallic materials but also of various types of composites [21], making them suitable for a wide range of applications.

Recent advancements in the field of interfaces and joints focus on a number of aspects, including processing techniques, product innovations, surface treatments, and the investigation of critical properties [22,23,24]. Products in which interfaces are combined using various types of joints are widely used in the automotive, transport, sports, shipbuilding, medical, and engineering industries, among others [25,26,27,28,29,30]. Advances in joint manufacturing technology continue to improve mechanical properties, but also biodegradability and recyclability [31,32]. Nanotechnologies and material modification techniques are being researched in order to further increase the strength, thermal stability, and fire resistance of their joints [33]. All this highlights the importance of interfaces and their joints today, and that further extensive research and modeling in this area is necessary. A certain amount of this research is presented in the Special Issue of *Materials* entitled “Computational and Experimental Modeling of Interfaces and Joints in Advanced Materials”, the introduction to which is this Editorial.

## 2. Overview of Papers Published in the Special Issue

As key components of offshore wind turbine systems [34], pitch bearings require exceptional resistance to material fatigue to ensure the extended service life and structural reliability demanded in a marine environment. Failure of these bearings caused by factors such as material fatigue due to rolling contact [35] or frictional phenomena [36] can seriously affect the economic efficiency of offshore wind turbines and potentially lead to accidents that threaten the safety of people and machinery. With this in mind, He et al. (Contribution 1) developed a simulation model of the pitch bearings used in a 3 MW offshore wind turbine to investigate the behavior of rolling contact fatigue under operating conditions based on continuous damage mechanics. In the damage process, they took into account both elastic and plastic damage. The results indicate that fatigue damage to the raceway shows significant nonlinearity, with elastic damage playing a dominant role in determining its fatigue life under operating conditions.

In the case of bolted flange joints, it is important to ensure that the joint is sufficiently tight [37,38]. Research on this topic was conducted by Jaszak and Grzejda (Contribution 2), who examined the influence of the symmetrical and asymmetrical shape of the core of a semi-metallic gasket on the tightness of bolted flange joints. Based on numerical calculations and experimental tests, they demonstrated that the asymmetrical core of the gasket provides a higher strain on the sealing graphite layer and leads to a more even distribution of strain on the individual ridges of the core. In addition, the leakage rate of the asymmetrical gasket was reduced by approximately 60% compared to the symmetrical gasket. It was also observed that the uniformity of pressure and strain distribution in the asymmetrical core gasket occurs across approximately 80% of the gasket width. The effect of reducing leakage in a flange joint sealed with an asymmetrical core gasket has been explained theoretically. It was shown that the main leakage flows through the porous structure of the graphite layer, while the leakage path at the interface between the rough metal profile and the graphite layer is several orders of magnitude lower.

Estimating the mechanical behavior of components to predict fatigue life using the Finite Element Method (FEM) [39] can be time-consuming, especially due to material nonlinearities. To address this, Marković et al. (Contribution 3) introduced a surrogate model using an Artificial Neural Network (ANN) [40] that allows the fatigue life of a component to be estimated quickly and directly based on its geometry, material properties, and loading conditions. For the purposes of ANN training, a non-parametric computational model based on FEM was developed for samples of components, surface-hardened and through-hardened, providing the necessary data. Simulations were performed for a wide range of geometric configurations, including different types and numbers of stress concentrators and different material conditions. To evaluate the generalization ability of the ANN model, it was tested on an independent data set generated from a parameter space different from that used for training. ANN showed satisfactory overall performance, with approximately 85% of data points falling within the constructed range (for a fatigue life factor of 3).

The torsional stiffness of rehabilitation robot joints is a key factor determining their performance, significantly affecting movement accuracy, stability, and user comfort. Keeping this in mind, Zou et al. (Contribution 4) presented a comprehensive analysis of the torsional stiffness of advanced traction drive joints based on fractal theory [41]. The research was conducted on an innovative traction drive mechanism that transmits torque using friction forces, eliminating the problems associated with mechanical shocks found in traditional gear transmissions. The surface topography was characterized using the Weierstrass–Mandelbrot function [42]. To obtain the tangential stiffness of individual contact pairs, a contact mechanics model was developed that takes into account the elastic–plastic deformation of micro-irregularities. It was shown that the stiffness of such a contact increases with normal load, contact length, and radius, while it decreases with tangential load and roughness parameter. The overall stiffness of the system shows a similar dependence on parameters, with a slight decrease with increasing output load when sufficient preload is applied. It has been confirmed that the fractal-based model allows for more accurate prediction of stiffness and provides valuable theoretical information.

The surface quality of biomedical implants, such as shoulder joint cups, plays a key role in their performance, durability, and biocompatibility. Most biomedical shoulder joints do not achieve optimal functionality when machined using conventional techniques such as grinding and lapping due to the inability to achieve nanometer-level smoothness, resulting in increased wear and friction, as well as potential failure. Advanced magnetorheological finishing (MRF) technology [43,44] provides improved surface quality through precise dimensional control during material removal. The effect of MRF on surface roughness, microhardness properties, and wear resistance of cobalt–chromium alloy shoulder joint caps, which are characterized by biocompatibility, was evaluated by Singh et al. (Contribution 5). In the study, they used an MRF system consisting of an electromagnetic tool to improve surface roughness from 0.35 µm to 0.03 µm. Microhardness measurements showed that the use of MRF leads to an increase from HV 510 to HV 560, which increases the wear resistance of the samples. After MRF, the coefficient of friction decreased from 0.12 to 0.06, demonstrating an improvement in the tribological properties of these implants. The results show that MRF technology offers excellent benefits in biomedical applications, as it extends the service life of implants and reduces medical complications, leading to better health outcomes for patients.

In recent years, rapid prototyping methods [45] have been gaining popularity in various industries. Among these methods, 3D printing technology is increasingly used for prototype production, mainly due to its relatively low development and production costs and the short time required for physical production. An example of the application of rapid prototyping techniques to the testing of jet nozzles was described by Madejski et al. (Contribution 6). They proposed integrating 3D printing methods with the results of experiments. Four different nozzle shapes were tested. Computational Fluid Dynamics (CFD) [46] was combined with 3D printing to provide quick responses and enable comparison of proposed solutions. A new evaluation criteria coefficient was proposed to calculate the production efficiency of nozzles using the printing method.

The mechanical and tribological properties of interfaces are influenced by, among other things, xenon ion irradiation [47,48]. This issue is addressed in the publication by Budzyński et al. (Contribution 7), which presents the results of research on the effect of irradiation with swift xenon ions on the properties of the Nimonic 90 superalloy [49]. Studies of irradiated samples of this alloy revealed significant changes in the crystal structure of the material, including the formation of new phases and partial amorphization of the surface layer, particularly evident at the highest irradiation fluence. Measurements of microhardness, friction coefficient, and wear showed a deterioration in the mechanical and tribological properties of samples irradiated with fluences of 1.0 × 10^14^ Xe^24+^/cm^2^ and 2.5 × 10^14^ Xe^24+^/cm^2^, which was attributed to the formation of radiation-induced defects. Increased friction and wear were observed at depths greater than the predicted range of xenon ions, indicating the occurrence of a long-range effect. After irradiation with a fluence of 5.0 × 10^14^ Xe^24+^/cm^2^, a radiation annealing effect was observed, which led to a partial reduction in previous damage, improved microhardness, and reduced wear. Thus, it was demonstrated that the use of Nimonic 90 superalloy in environments exposed to intense ionizing radiation, such as nuclear reactors, should be limited.

When determining the effective elastic properties of composite materials, it is necessary to follow the principles of homogenization theory [50,51]. A novel strategy in the theory of elastic flat composites was presented by Czapla et al. (Contribution 8). It involves quantifying macroscopic properties and designing an analytical–numerical algorithm for deriving expressions for effective constants. The focus in this case is on the effective elastic constants of dispersed random composites. The concept of a Representative Volume Element (RVE) [52] has been reanalyzed and revised. It has been rigorously demonstrated that the RVE must be a fundamental domain of a two-dimensional torus. Effective tensors of elasticity theory undergo decomposition into geometric and physical parts, represented by structural sums and material constants of components. An innovative computational methodology based on such decomposition has been applied to a two-phase isotropic composite with non-overlapping circular inclusions embedded in an elastic matrix. It has been shown how effective tensors depend on geometric distributions of inclusions and the computational protocols used. In the methodology, analytical approximate formulas for the effective tensor defining the effective shear modulus for low inclusion concentrations are transformed using resummation methods into practical expressions valid for all inclusion concentrations. In contrast, the critical index for the effective shear modulus is calculated based on polynomials derived for the modulus.

A continuation of the analysis contained in Czapla et al. (Contribution 8) is included in Mityushev et al. (Contribution 9). It consists of extending the analytical study by incorporating machine learning (ML) methods [53,54] for the quantitative classification of microstructures. The methodology is based on decomposing expressions for effective tensors into geometric and physical parts, represented by structural sums [55] and physical constants specific to individual components. The study concerns a two-phase composite with non-overlapping circular inclusions embedded in an isotropic elastic matrix. The random distribution of inclusions ensures the macroscopic isotropy of the composite. The key result is a clear demonstration of how the effective tensor depends on the geometric probability distributions of the inclusions and the computational protocols used in their implementation. These steps constitute a strategy for investigating elastic fiber composites [56,57], classifying them according to macroscopic properties, and describing an analytical algorithm for deriving expressions for calculating effective constants. The decomposition theorem and the construction of feature vectors consisting of structural sums were used as input data for ML analysis. As a result, a computationally efficient strategy has been developed for classifying dispersed random composites that cannot be distinguished based on simple observations [58].

The results of experimental studies on a selected, preloaded asymmetric multi-bolted connection under operational conditions were presented by Grzejda et al. (Contribution 10). At the start of the tests, the bolts were preloaded in a predetermined optimal sequence in three stages, with the bolt forces monitored using a calibrated measurement system equipped with strain gauges [4,16]. The connection was then subjected to sinusoidal loads with amplitudes of 10 kN and 20 kN at various frequencies. The obtained data were collected and recorded using the prepared measurement pathway components and then processed in MATLAB/Simulink R2018b [59,60]. The observed measurement results were presented in graphs showing the distribution of forces in the bolts as a function of cyclically varying working loads. It has been shown that the forces in the bolts also vary cyclically relative to their initial preload values. Due to the asymmetry of the connection, this variation differed for individual bolts. The results presented in this paper supplement the database of experimental results, which will be used in the future to verify a systemic approach to modeling multi-bolted connections.

## 3. Conclusions

The collection of articles in this Special Issue constitutes a comprehensive and future-oriented study devoted to computational and experimental modeling of interfaces and joints in advanced materials currently used in mechanical engineering.

Several studies (Contribution 1 and Contribution 3) focus on the behavior of working surfaces under operational conditions caused by fatigue. They used simulation models to estimate the fatigue life of the components under investigation.

Research into the surface quality of joints in biomedical implants (Contribution 5) advocates the use of advanced MRF technology. This technology makes it possible to achieve better tribological properties in these implants and enhance their wear resistance. Conversely, the mechanical and tribological properties of interfaces are adversely affected by, among other things, xenon ion irradiation (Contribution 7).

A fractal-based model allows for more accurate prediction of the torsional stiffness of the joints in the traction drive system of rehabilitation robots (Contribution 4). The contact stiffness of the components connected in such a joint increases with normal load, contact length, and radius, while it decreases with tangential load and roughness.

The results of experimental tests on preloaded, externally loaded multi-bolted flanged connections were described in Contribution 2 and Contribution 10. Compared to a symmetric gasket core, an asymmetric gasket core results in a more even distribution of deformation across the individual ridges of the core and provides better sealing performance. To ensure the tightness of this type of joint, it is also essential to maintain the proper preload on the bolts.

## Data Availability

The data presented in this study are available on request from the author.

## Abbreviations

The following abbreviations are used in this paper:

ANN	Artificial Neural Network
CFD	Computational Fluid Dynamics
FEM	Finite Element Method
ML	Machine learning
MRF	Magnetorheological finishing
RVE	Representative Volume Element
